# To Stop Coughing

**Published:** 1866-05

**Authors:** 


### "TO STOP COUGHING.

"Slight irritation of the throat may be relieved by sipping a little slippery elm tea, or by suckling a piece of gum arabic. These articles coat over the mucus membrane and prevent the irritation of the air. A very few drops of paregoric held in the mouth, and allowed to trickle down the throat, will allay coughing. The best cough medicine for children, one which we have used for several years with entire satisfaction, is the following: Mix in a phial equal parts of paregoric, castor oil and syrup of ipecac. Always shake well just before using. A few drops of this swallowed, but not washed down by water or other fluid, will almost always soothe a cough. Repeat the dose as often as the cough returns. From one-fourth to one-half a teaspoonful may be given when a lesser quantity does not suffice. A large dose after a full meal may produce a little nausea. Children who are subject to coughs should eat very little supper, and indeed, all children should eat much less and simpler food at night than at morning or noon. The above mixture may be kept on hand ready prepared, as it does not deteriorate if kept corked. It may interest those afraid of mineral medicines (though they partake freely of common salt, which is a mineral,) to know that the ingredients are all 'vegetable.'"

The above is going the rounds, as credited to "Hall's Journal of Health;" although wrongfully, yet as nothing is so bad but that some good use may be made of it, so this occasion is taken to impress some wholesome truths upon our readers, and which are literally of vital importance.

If there is one truth more than another persistently taught in these pages, it is that it is generally dangerous and always injurious to "stop a cough." When a man has consumption, and his cough is stopped by anything he swallows, or if it suddenly stops itself, he will die in a week, because cough is nature's means of bringing the phlegm up from the lungs; all consumptives will testify that the more freely they can "bring up," the better they feel, simply because what is brought up comes from the lungs, leaves more room for air; they breathe freer and fuller. The cough brings the matter from the lungs to the top of the throat; from that point it is brought with a hack or a hem, into the mouth, from which it is passed out by the act of expectoration; if there was no cough, there would be an inevitable accumulation in the lungs, until they would be filled with yellow matter; no air could penetrate, and death would necessarily ensue. Merely "stopping" a cough, which is the effect aimed at by all medicines sold for coughs, colds and consumptions, not only does nothing towards effecting a cure, but counteracts nature in her efforts to do the same, hence tend to destroy, instead of preserve: of all the medicines known, and which are given in reference to coughs, opium is the most pernicious ultimately; whether it be in the shape of the crude material itself, or paregoric, or laudnam or morphine; because, if it alone is relied upon, it is like preventing the appearance of smoke on board ship, while the hold remains on fire, and is every moment in process of destruction. When these medicines are ignorantly given to children for cough, or pain, or bowel complaint, they have an immediate but deceptive good effect, to be followed with convulsions or water on the brain; this accounts for the thousands of deaths of young children in summer time by fits and convulsions.

Let it be remembered by our readers that we have steadily aimed to avoid giving medical prescriptions in the pages of this Journal, only making an exception in case of cholera, and then merely for the purpose of arresting the progress of the disease, until a physician could be secured. When a person is sick and actually needs medicine, he should send for a physician, he ought no more to medicate himself than to mend his watch, or repair an old shoe. Physicians themselves after half a century's experience, sometimes mistake the meanings of a symptom, and a mistake in certain cases, is death. The constant aim of this Journal is two-fold. First, to show how to avoid sickness; second, to teach what may be done towards restoration by prompt attention, good nursing and the use of diet, exercise and air; but if more is needed, by all means send for a resident physician.

